# An Experimental and Theoretical Investigation of 1-Butanol Pyrolysis

**DOI:** 10.3389/fchem.2019.00326

**Published:** 2019-05-14

**Authors:** Marzio Rosi, Dimitris Skouteris, Nadia Balucani, Caterina Nappi, Noelia Faginas Lago, Leonardo Pacifici, Stefano Falcinelli, Domenico Stranges

**Affiliations:** ^1^Department of Civil and Environmental Engineering, University of Perugia, Perugia, Italy; ^2^Master-Up, Perugia, Italy; ^3^Laboratory of Molecular Processes in Combustion, Department of Chemistry, Biology and Biotechnologies, University of Perugia, Perugia, Italy; ^4^Department of Chemistry, University of Rome “La Sapienza”, Rome, Italy

**Keywords:** combustion chemistry, pyrolysis, biofuels, ab initio calculations, rate constants

## Abstract

Bioalcohols are a promising family of biofuels. Among them, 1-butanol has a strong potential as a substitute for petrol. In this manuscript, we report on a theoretical and experimental characterization of 1-butanol thermal decomposition, a very important process in the 1-butanol combustion at high temperatures. Advantage has been taken of a flash pyrolysis experimental set-up with mass spectrometric detection, in which the brief residence time of the pyrolyzing mixture inside a short, resistively heated SiC tube allows the identification of the primary products of the decomposing species, limiting secondary processes. Dedicated electronic structure calculations of the relevant potential energy surface have also been performed and RRKM estimates of the rate coefficients and product branching ratios up to 2,000 K are provided. Both electronic structure and RRKM calculations are in line with previous determinations. According to the present study, the H_2_O elimination channel leading to 1-butene is more important than previously believed. In addition to that, we provide experimental evidence that butanal formation by H_2_ elimination is not a primary decomposition route. Finally, we have experimental evidence of a small yield of the CH_3_ elimination channel.

## Introduction

Combustion is a complex phenomenon that involves physical and chemical processes. Because of its importance in many human activities, a thorough characterization has been pursued for decades aiming at optimizing the performance of engines or heaters or other devices exploiting combustion systems (Gardiner, 2000). More recently, the recognition that pollutants are massively emitted from flames has stimulated a more accurate characterization of the combustion environments trying to reduce or minimize those harmful emissions (see, for instance, Battin-Leclerc et al., 2013 and references therein). Since pollutants are normally produced in trace amounts, an in-depth characterization is necessary that requires a chemical description of the elementary reactions occurring in flames at the atomistic level (see, for instance, Balucani et al., 2012a). As a result of such a cumulative effort, we have now a good knowledge of the elementary reactions lying at the heart of combustion of fossil fuels, although several problems remain (e.g., soot formation, NO formation). In the last decades, the advent of biofuels caused by the expected shortage of non-renewable fuels and by the search of carbon-neutral fuels has posed a new challenge to the chemical characterization of combustions as, typically, biofuels are constituted by more complex molecules than traditional fuels (Kohse-Höinghaus et al., 2010). Remarkably, most of the proposed biofuels are O-bearing organic compounds like alcohols, ethers and esters (Kohse-Höinghaus et al., 2010). While the use of biofuels seems to reduce the production of several pollutants which are typical of fossil fuels, their combustion generates a new group of pollutants which are instead absent in the case of fossil fuels, namely highly toxic aldehydes and ketones (Kohse-Höinghaus et al., 2010; Battin-Leclerc et al., 2013). Therefore, the work done for the characterization of fossil fuels combustion must be extended to the new fuels.

Among biofuels, a promising family of compounds are bio-alcohols, which have been already used in several countries. In particular, 1-butanol is considered an excellent candidate for massive use, because of its high energy content, low water absorption, high miscibility with conventional fuels, and the possibility of being used in conventional engines (Kohse-Höinghaus et al., 2010). For this reason, 1-butanol combustion has been widely investigated and the global combustion properties, such as heat release and CO_2_ emission, have been characterized (see, for instance Dagaut et al., 2009; Sarathy et al., 2009, 2012, 2014; Black et al., 2010; Grana et al., 2010; Harper et al., 2011; Cai et al., 2012; Yasunaga et al., 2012; Feng et al., 2017). Among the various elementary processes occurring when burning 1-butanol, its unimolecular high temperature decomposition is certainly important because of the high temperature conditions of combustion environments (Grana et al., 2010; Harper et al., 2011; Cai et al., 2012; Karwat et al., 2015). This has motivated numerous experimental and theoretical studies on the pyrolysis of butanol (see Cai et al., 2012, and references therein). The most comprehensive study is that by Cai et al. (2012) where a combined experimental and theoretical approach has been used. More precisely, rate coefficients of unimolecular reactions were calculated with the variable reaction coordinate-transition-state theory (VRC-TST) and the Rice-Ramsperger-Kassel-Marcus (RRKM) theory on a newly derived potential energy surface while the experiments were performed in a flow reactor at a pressure ranging from 5 to 760 Torr with synchrotron VUV (Vacuum Ultra-Violet) photoionization mass spectrometry to identify the pyrolysis products. In spite of the use of such a sophisticated experimental technique, a model needed to be used to interpret the experimental results and it remained unclear whether some observed species are primary products of the unimolecular decomposition of 1-butanol or secondary products formed in the flow reactor. In the Laboratory of Molecular Processes in Combustion of the University of Perugia, we are now building an apparatus to perform experiments based on the flash pyrolysis technique and mass spectrometric detection by FUV (Far Ultra-Violet) ionization at 118.2 nm within the frame of the AMIS project (http://amis.chm.unipg.it). The same approach has already used by Chambreau et al. (2000), Chambreau et al. (2005), Weber et al. (2006), Morton et al. (2011), and Liu et al. (2018), while flash pyrolysis coupled to other detection techniques has been implemented by O'Keeffe et al. (2008), Vasiliou et al. (2009, 2011), Scheer et al. (2011, 2012), Urness et al. (2013), Prozument et al. (2014), Buckingham et al. (2016), Holzmeier et al. (2016), Porterfield et al. (2017), Vasiliou et al. (2017), Zhao et al. (2017), Abeysekera et al. (2018), and Ormond et al. (2018). The advantages of the flash pyrolysis technique is that the very limited residence time inside a SiC tube of ca. 2–2.5 cm, which can be resistively heated at temperatures as high as 1,500 K, allows one to better define the yield of the primary pyrolysis products (Guan et al., 2014; Weddle et al., 2018; Zagidullin et al., 2018) because the probability of secondary reactions involving pyrolysis products or even further pyrolysis of primary products are strongly reduced (Guan et al., 2014).

In this work, we report a test-case we have performed at the University of Rome where an apparatus exploiting flash pyrolysis with traditional electron impact mass spectrometry is operative (O'Keeffe et al., 2008). The apparatus has been used in the past to characterize the flash pyrolysis of azidoacetone, while its main applications have focused on the UV photodissociation of radicals produced by the pyrolysis of a suitable precursors (Stranges et al., 1998, 2008; Chen et al., 2010, 2011; Stranges and Ripani, 2012). Because of its relevance and relative simplicity, our test-case involved the flash-pyrolysis of 1-butanol. To the best of our knowledge this is the first experiment on the flash pyrolysis of 1-butanol. New physical insights emerged from this study concerning the importance of H_2_O-elimination and the possibility that 1-butanal is a primary product. We have also completed this study by performing dedicated new electronic structure calculations at the B3LYP and CCSD(T) levels of theory of the stationary points necessary to describe the unimolecular decomposition of 1-butanol. We have also calculated the energetics of previously ignored decomposition pathways involving the rupture of C-H and O-H bonds. These data have been used to perform RRKM estimates under the conditions of our experiments. A more detailed picture of the thermal decomposition of 1-butanol has emerged.

## Experimental Method

**Figure 1:**
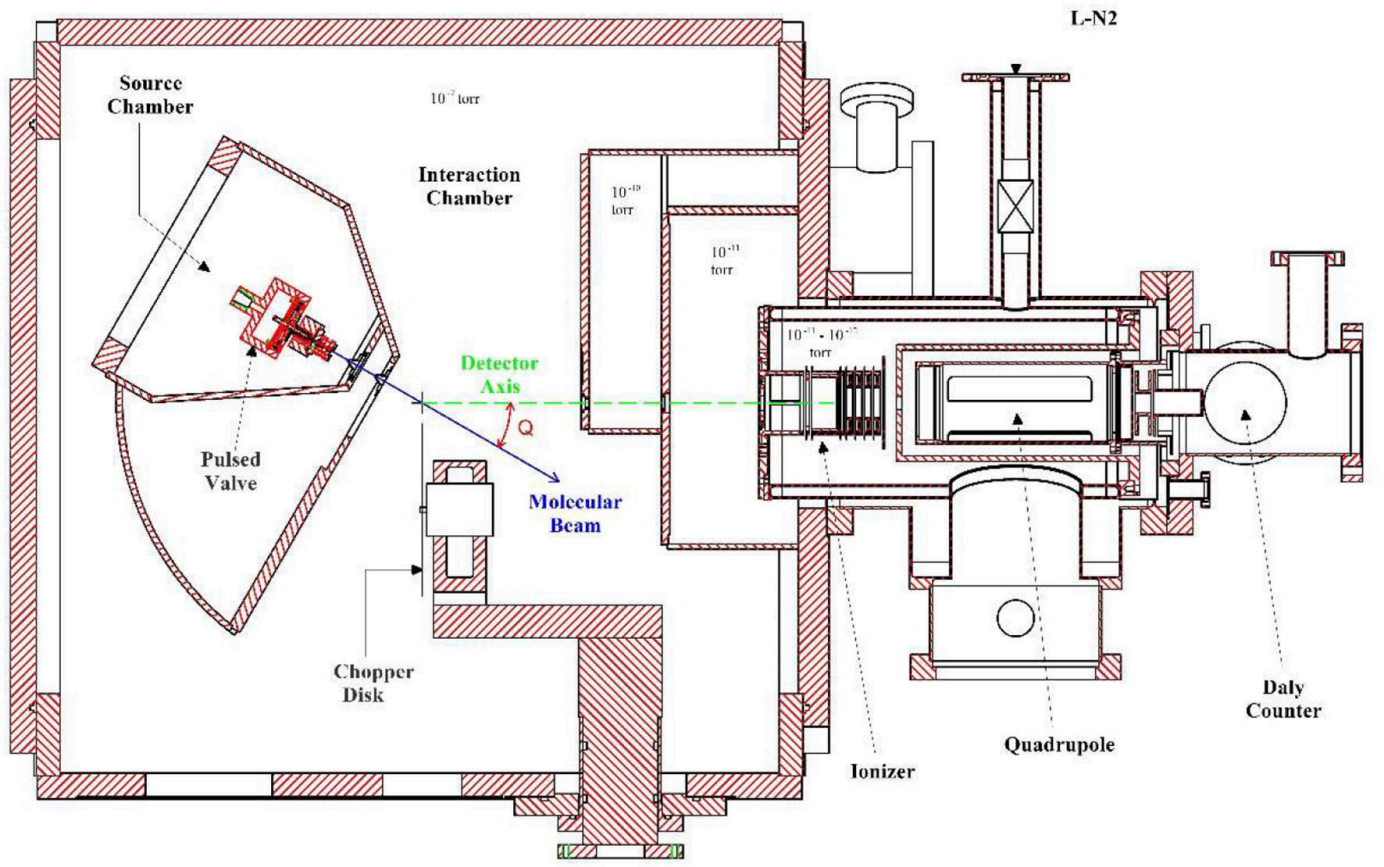
Cross section of the Rotating Source Photofragment-Translational spectrometer (at University of Rome “La Sapienza”).

The experimental measurements in this work were carried out on a rotating source photofragment-translational spectrometer (at University of Rome “La Sapienza”) which is normally utilized to study UV photodissociation processes. A drawing of this apparatus is shown in Figure 1. When it is utilized for pyrolysis experiments the source chamber is positioned at 0° so that the molecular beam direction is along the detector axis.

**Figure 2:**
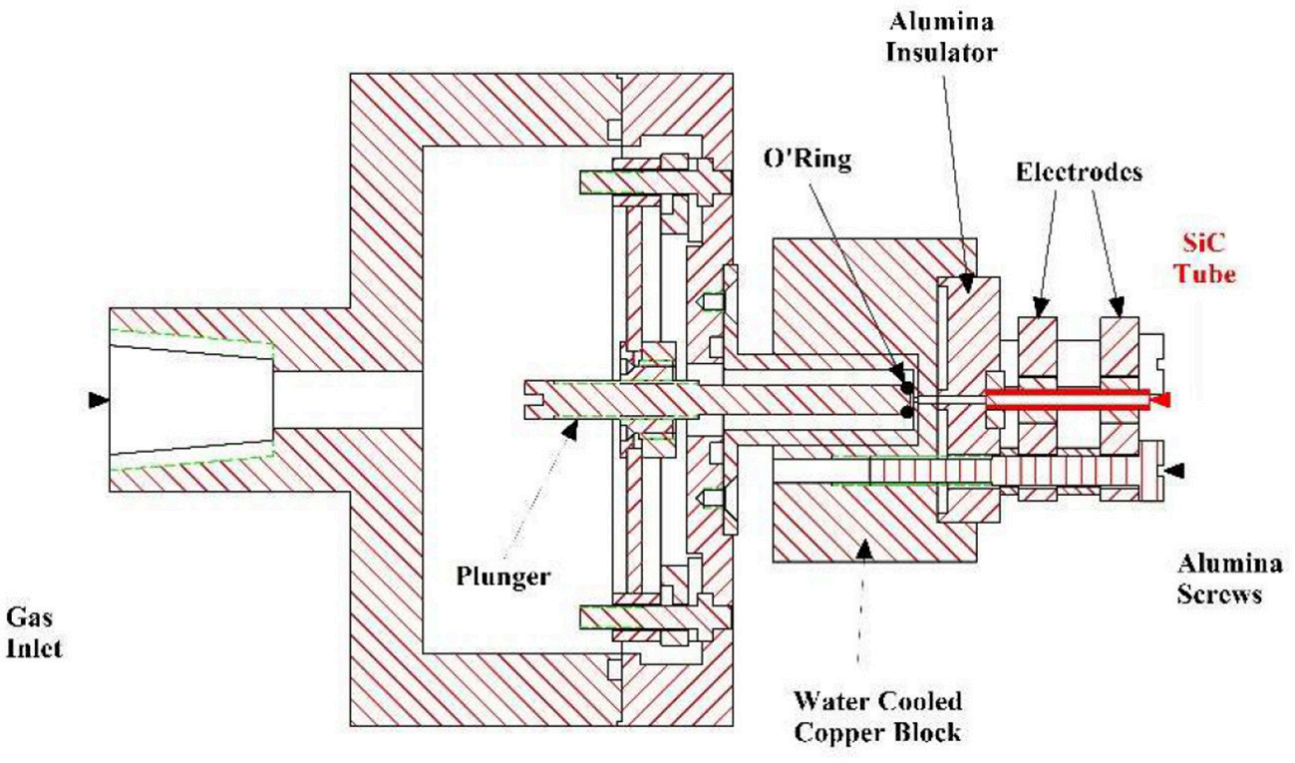
Cross section of the “flash-pyrolytic” source coupled with the pulsed valve.

A 0.2% mixture of 1-butanol seeded in Ar was sent to a piezoelectrically activated pulsed valve (Proch and Trickl (1989) operated at a repetition rate of 100 Hz. This pulsed valve was coupled to a “flash-pyrolytic” source, already utilized to generate molecular beams of hydrocarbon free radicals (Stranges et al., 2008; Chen et al., 2011), and a drawing is reported in Figure 2.

The gas mixture (1.3 bar) was expanded through a resistively heated SiC tube (length 23 mm) inducing the thermal decomposition of the 1-butanol molecules. When the gas mixture exits the hot SiC tube it undergoes a supersonic expansion, resulting in internal cooling of the thermal decomposition products, which are thus stable to further dissociation during their flight time to the electron impact ionizer. This aspect gives a big advantage to this technique as compared to conventional pyrolysis methods. It is due to the short residence time of the parent molecule in the pyrolysis zone (20–30 μs) together with the internal cooling of the nascent thermal products by the supersonic expansion. Therefore, in the case of pyrolysis of organic molecules, which proceeds via stepwise mechanisms, it is possible to isolate intermediate thermal decomposition products that are not observed using conventional oven techniques, providing a more detailed insight into the mechanisms involved.

The neutral molecules fly along the detector axis for 40 cm before reaching the ionizer where they are ionized by electron impact (70 and 100 eV electron energy), then the ions are mass selected by a quadrupole mass filter (Extrel 150QC) and counted by a Daly type counter. In order to minimize the background signal, mainly due to the residual gas, the surfaces of the ionizer region are cooled with liquid nitrogen allowing to reach a pressure of <1.0 × 10^−11^ mbar.

Time-of-Flight (ToF) spectra at different mass to charge ratios, m/e, were recorded as a function of the electrical power dissipated through the SiC tube, by inserting a spinning slotted disk (200 Hz) into the molecular beam to select a “slice” (~7.5 μs) of the gas pulse. These spectra were recorded and stored on a computer by using a Multi Channel Scaler (MCS-pci, EG&G Ortec). In order to obtain the mass spectra of the 1-butanol pyrolysis products it was preferred to integrate the ToF spectra recorded for different masses, by interrogating a very small part (~7.5 μs) of the gas pulse, to obtain background modulation and subtraction. The power dissipated through the SiC tube was converted into temperature by measuring the beam velocity of a neat He beam at the same experimental conditions. The velocity of a supersonic ideal atomic beam is related to the temperature of the source (SiC tube) via the expression $V=\sqrt{\frac{5RT}{m}}$, where *T* is the source temperature, *m* is the mass of the gas, *V* is the atomic beam velocity, and *R* is the gas constant.

## Theoretical Methods

The potential energy surface of the species of interest was calculated employing a computational strategy which has already been utilized with success in several cases (see, for instance Leonori et al., 2009a,b; de Petris et al., 2011; Rosi et al., 2012; Skouteris et al., 2015; Troiani et al., 2017). In this scheme the lowest stationary points were optimized at the B3LYP (Becke, 1993; Stephens et al., 1994) level of theory in conjunction with the correlation consistent valence polarized set aug-cc-pVTZ (Dunning, 1989; Kendall et al., 1992). At the same level of theory we have computed the harmonic vibrational frequencies in order to check the nature of the stationary points, i.e., minimum if all the frequencies are real, saddle point if there is one, and only one, imaginary frequency. The assignment of the saddle points was performed using intrinsic reaction coordinate (IRC) calculations (Gonzalez and Schlegel, 1989, 1990). The energy of the main stationary points was computed also at the higher level of calculation CCSD(T) (Bartlett, 1981; Raghavachari et al., 1989; Olsen et al., 1996) using the same basis set aug-cc-pVTZ. Both the B3LYP and the CCSD(T) energies were corrected to 298.15 K by adding the zero point energy and the thermal corrections computed using the harmonic vibrational frequencies evaluated at B3LYP/aug-cc-pVTZ level. Corrections to other temperatures were performed following the same procedure. For comparison purposes, some calculations were performed also at the Gaussian-3 (G3) (Curtiss et al., 1998) and Gaussian-3 using B3LYP structures and frequencies (G3B3) (Baboul et al., 1999) level. All calculations were done using Gaussian 09 (Frisch et al., 2009) while the analysis of the vibrational frequencies was performed using Molekel (Portmann and Lüthi, 2000;Flükiger et al., 2000-2002).

The rate constants for each unimolecular reaction in our scheme were calculated as a function of energy using the RRKM scheme (as in our previous works, see Balucani et al., 2009, 2010, 2012b; Leonori et al., 2009a, 2013), through the expression

$$\begin{array}{l} k\left(E\right)=\frac{N\left(E\right)}{h\;\rho \left(E\right)} \end{array}$$

where *N(E)* is the sum of states of the transition state, ρ*(E)* is the density of states of the reactant and *h* is Planck's constant. For the calculation of each density of states the rigid rotor/harmonic oscillator model was assumed. Even though the treatment of low-frequency modes as harmonic can lead to errors, we have chosen this strategy as the best alternative so as to keep the vibrational modes uncoupled and to avoid any arbitrariness in hindered rotor potentials. Moreover, tunneling was taken into account using the imaginary frequency of each transition state and modeling the potential energy as an Eckart barrier with the correct height (as calculated from the energy) and width (as calculated from the imaginary frequency). The RRKM code used is a home-made one developed by one of the authors and previously used in many systems.

In this way, for each unimolecular reaction in our scheme, we generate energy-dependent rate constants k(E) for a fixed range of energies, ranging from the lowest useful energy (the zero-point energy of the 1-butanol species) up to 1,500 kJ mol^−1^. The hypothesis here is that, during the pyrolysis event, the energy of the molecule remains constant and statistically distributed among the degrees of freedom. Subsequently, for each product species, the corresponding energy-dependent rate constants were Boltzmann-averaged (using the density of states of 1-butanol and the corresponding partition function) to calculate temperature dependent rate constants up to 2,000 K for each species. Thus, we are assuming that, prior to the pyrolysis event, butanol molecules are thermalized by interaction with the environment.

Where no obvious transition state is indicated, we performed a variational transition state theory (VTST) calculation, calculating RRKM rate constants for a range of candidate transition states and choosing the minimum one. A full electronic calculation was performed for each candidate transition state, with no use of Lennard-Jones potentials for the long-range interactions.

## Experimental Results

**Figure 3:**
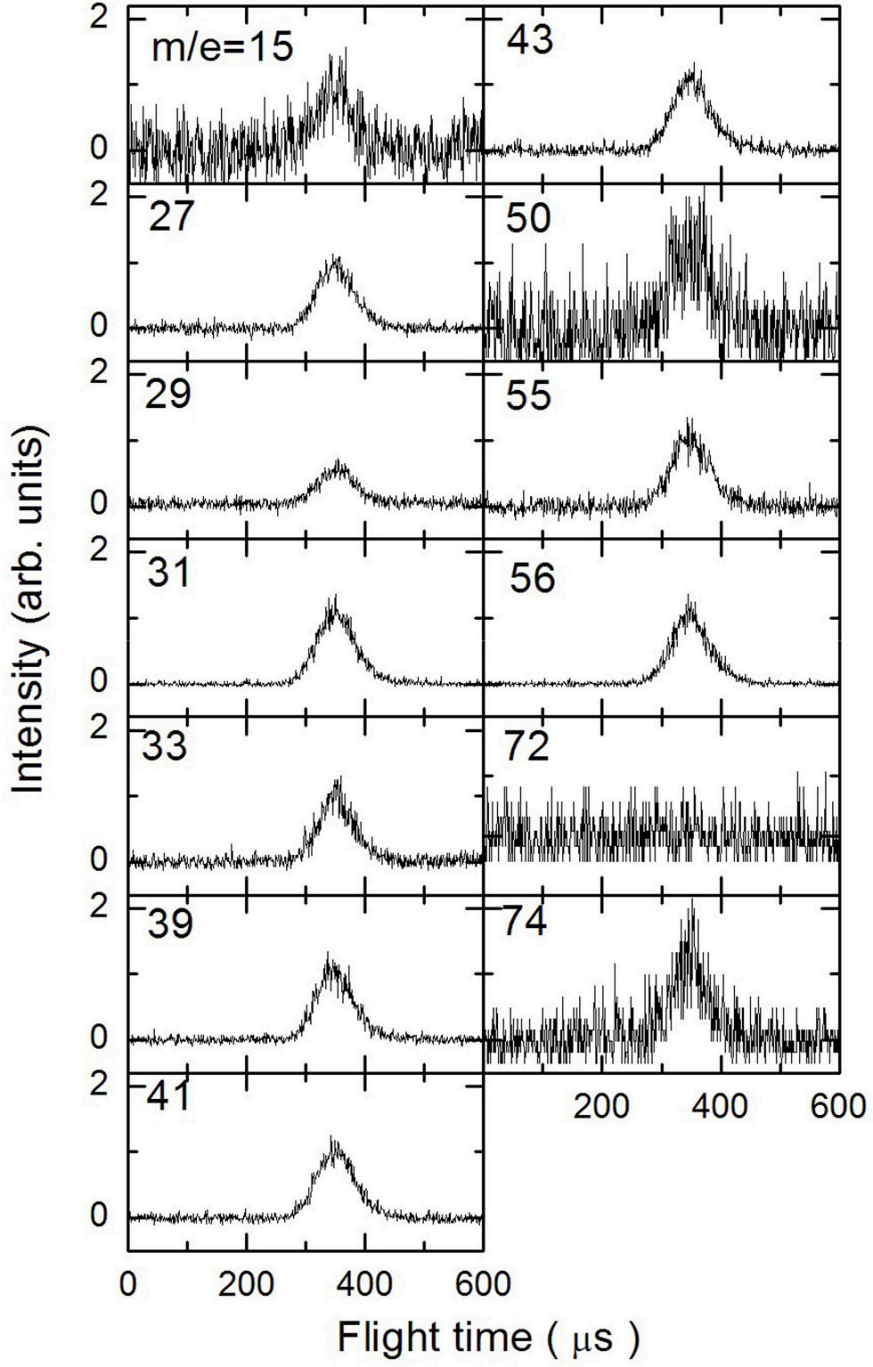
Time of Flight spectra recorded at a temperature of 997 K for several mass-to-charge ratios. Each spectrum has been arbitrarily normalized after background subtraction.

**Figure 4:**
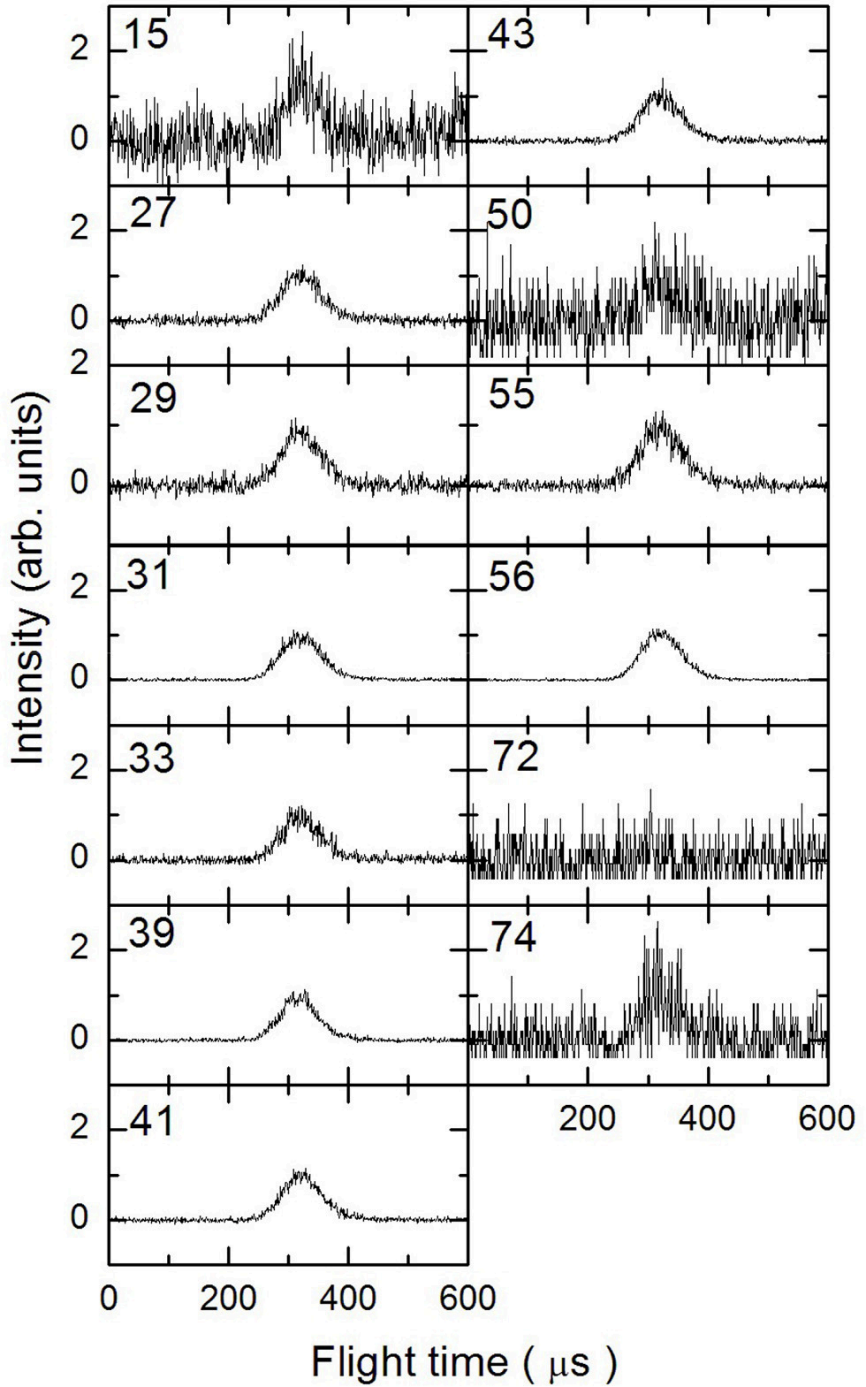
Time of Flight spectra recorded at a temperature of 1,214 K for several mass-to-charge ratios. Each spectrum has been arbitrarily normalized after background subtraction.

**Figure 5:**
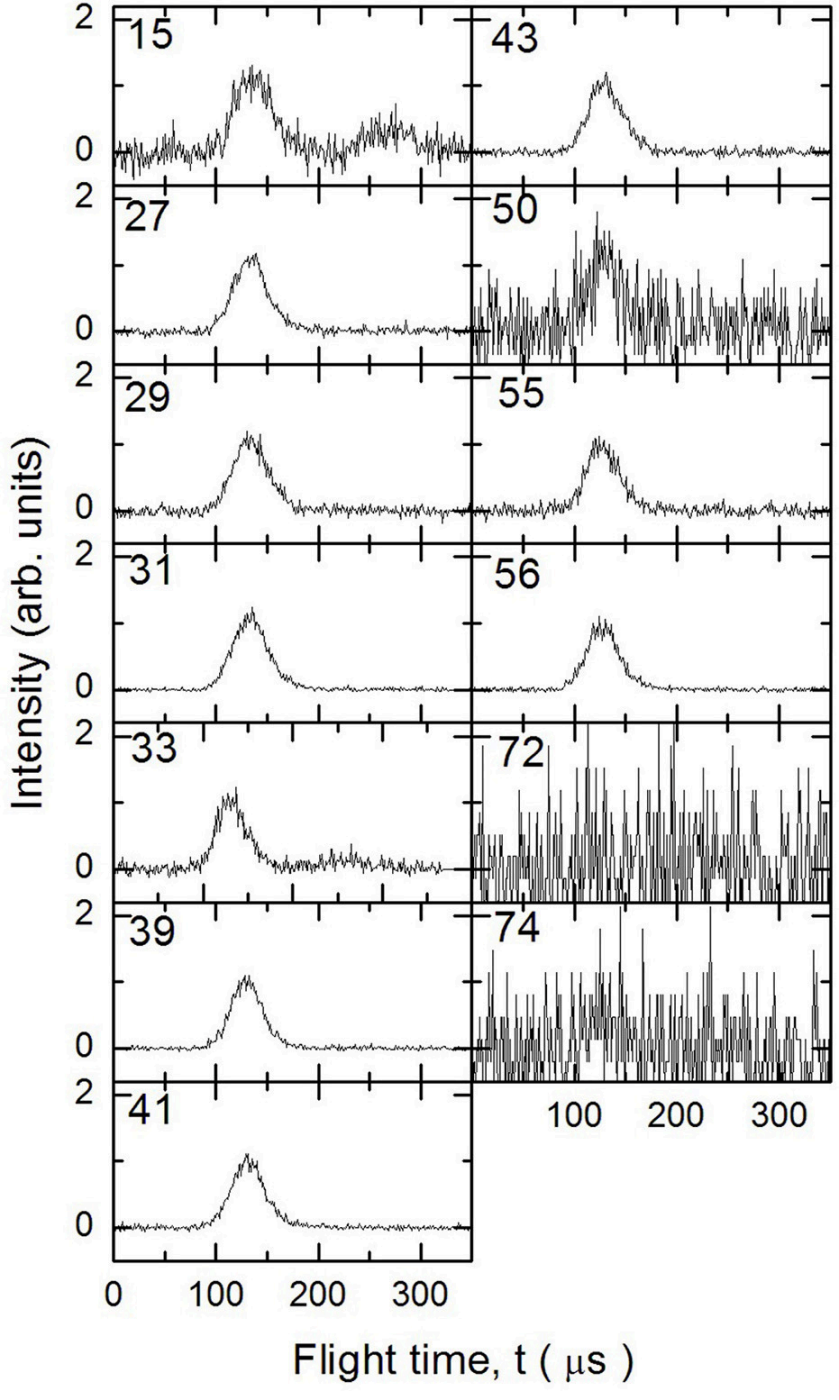
Time of Flight spectra recorded at a temperature of 1,355 K for several mass-to-charge ratios. Each spectrum has been arbitrarily normalized after background subtraction.

In Figures 3–5 are reported the ToF spectra recorded at several mass-to-charge ratios, m/e, at the temperatures of 997, 1,214, and 1,355 K. Other spectra were recorded at lower temperatures, but in those cases the mass spectra derived by ToF integration are in all aspects similar to the one derived for 1-butanol at 300 K. Therefore, in those conditions no pyrolysis occurred inside the SiC tube.

To analyze the data and derive the mass spectra of the pyrolysis products alone, the contribution deriving from the non-pyrolyzed 1-butanol must be subtracted. The procedure normally used consists in recording the mass spectrum of the molecule to be pyrolyzed at room temperature and then subtracting this spectrum from those recorded at different temperatures by normalizing their weight to the signal of the parent peak. In the case of 1-butanol, this procedure is not possible because, as often happens for alcohols, the parent peak has a very low intensity compared to all the other masses present in the spectrum.

The procedure followed to subtract this contribution is based on the consideration that in the ToF spectra recorded at different temperatures for masses 57 and 72, the signal is absent. In the butanal (CH_3_CH_2_CH_2_CHO) mass spectrum (NIST-1 Chemistry Webbook)^1^ the masses 72 and 57 are about 75 and 25% of the most intense peak (m/e = 44), respectively. This implies that the presence of butanal would be detectable even in small concentrations. We can therefore exclude the formation of CH_3_CH_2_CH_2_CHO and H_2_ from the pyrolysis of 1-butanol under our experimental conditions.

If we exclude the presence of H and OH elimination reactions, since they are very endothermic, then the signals at m/e 55 and 56 are due only to 1-butanol and 1-butene. To determine their contribution in the ToF spectra we took advantage of their quite different fragmentation pattern for the two masses (m56 = 100% and m55 = 30% for 1-butanol, m56 = 40% and m55 = 20% for 1-butene) which allow to set up the following system of two equations:

$$\begin{array}{l} \%\text{Py}56={\text{C}}_{1}\times \%\text{A}56+C_{2}\times \;\%\text{Bu}56\\ \%\text{Py}55={\text{C}}_{1}\times \%\text{A}55+C_{2}\times \;\%\text{Bu}55 \end{array}$$

%Py56 and %Py55 represent the percentages of these two masses in the pyrolysis spectra (normalized to 100 for the most intense mass), %A56 and%A55 are the relative abundances of the two masses in the mass spectrum of 1-butanol (NIST-2 Chemistry Webbook)^2^, %Bu56 and %Bu55 are the relative abundances of the two masses in the mass spectrum of 1-butene (NIST-3 Chemistry Webbook)^3^, and C_1_ and C_2_ represent the apparent percentages of 1-butanol and 1-butene, respectively, present in the molecular beam.

The mass spectra of neat 1-butanol and 1-butene were measured under the same experimental conditions as pyrolysis but with the SiC tube at room temperature and then subtracted from the pyrolysis mass spectra. In this way the mass spectra relative to the other dissociation channels with lighter products were obtained.

To obtain the branching ratio between 1-butene and 1-butanol, their apparent percentages have to be corrected for the different ionization cross section by electron impact: 11.74${\ddot{\text{A}}}^{2}$ for 1-butene (NIST-4 Chemistry Webbook)^4^ and 12.40${\ddot{\text{A}}}^{2}$ for 1-butanol (Hudson et al., 2003).

**Figure 6:**
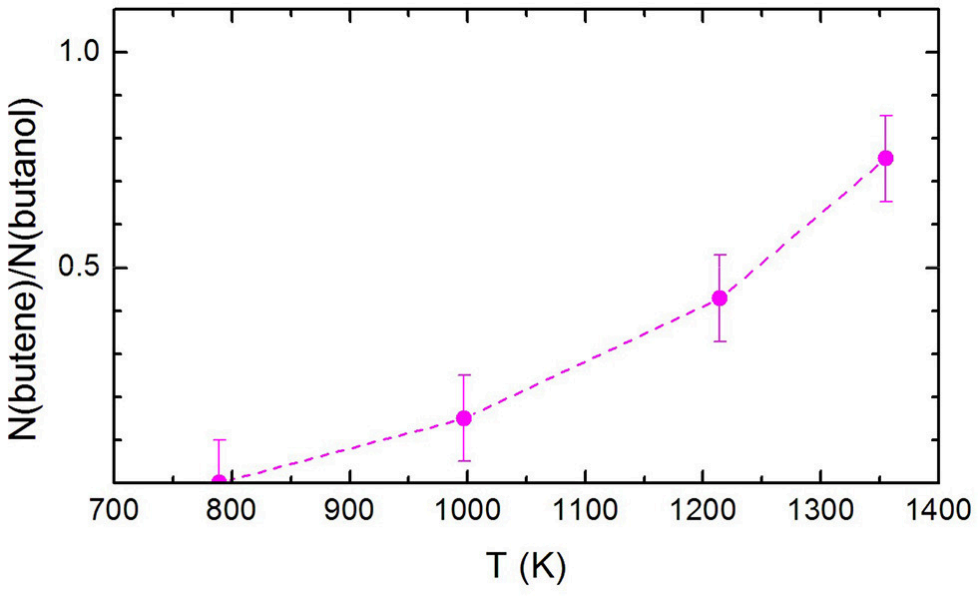
The ratio between the signal attributed to butene and the undissociated remaining 1-butanol, N(butene)/N(1-butanol), at the three temperatures investigated. The dashed line has been drawn to guide the eye. The estimated error of ±10% has also been indicated.

The ratio between the signal attributed to butene and the undissociated remaining 1-butanol, N(butene)/N(1-butanol), is shown in Figure 6. Remarkably, N(butene)/N(1-butanol) keep on increasing with the temperature. If we compare the present trend with the butene signal recorded by Cai et al. (2012), it is clear that there is no secondary pyrolysis of butene or secondary reactions affecting its abundance in the pyrolyzed mixture. This can be taken as a confirmation that the experimental method employed here allows a better characterization of the primary events in the pyrolysis.

**Figure 7:**
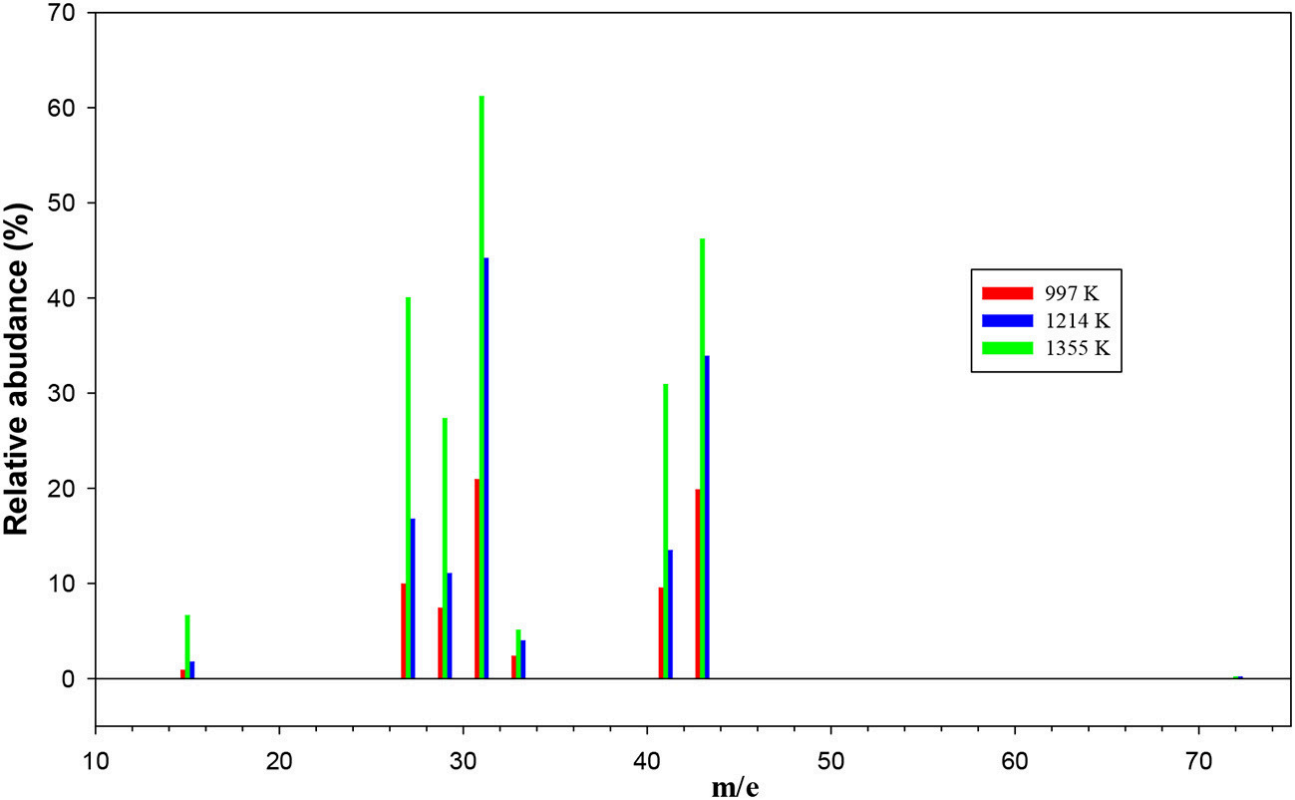
The remaining mass spectra at the three temperatures investigated once the contributions of undissociated 1-butanol and main pyrolysis product 1-butene have been subtracted.

The remaining mass spectra at the three temperatures investigated, once the contributions of undissociated 1-butanol and main pyrolysis product 1-butene have been subtracted, are reported in Figure 7. As clearly visible, signals at m/e 42, 42, 33, 31, 29, 27, and 15 have been recorded. They correspond to both parent ions of pyrolysis products and their daughter species. Even though it is not possible to disentangle them with the present results, we note that the intensity of all peaks is increasing, thus testifying that the extent of 1-butanol pyrolysis increases with the temperature for all species.

## Theoretical Results and Discussion

**Figure 8:**
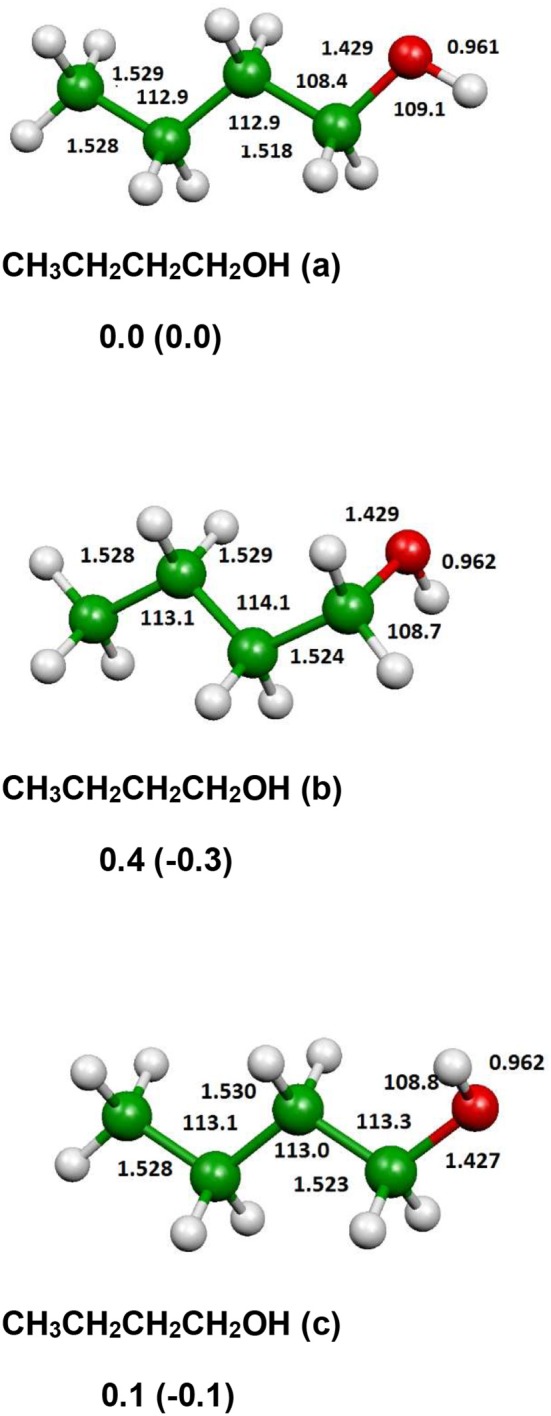
B3LYP optimized geometries (Å and °) and energies, relative to CH_3_CH_2_CH_2_CH_2_OH (a), (kJl/mol) at 298.15 K of minima of 1-butanol; CCSD(T) relative energies are reported in parentheses.

**Figure 9:**
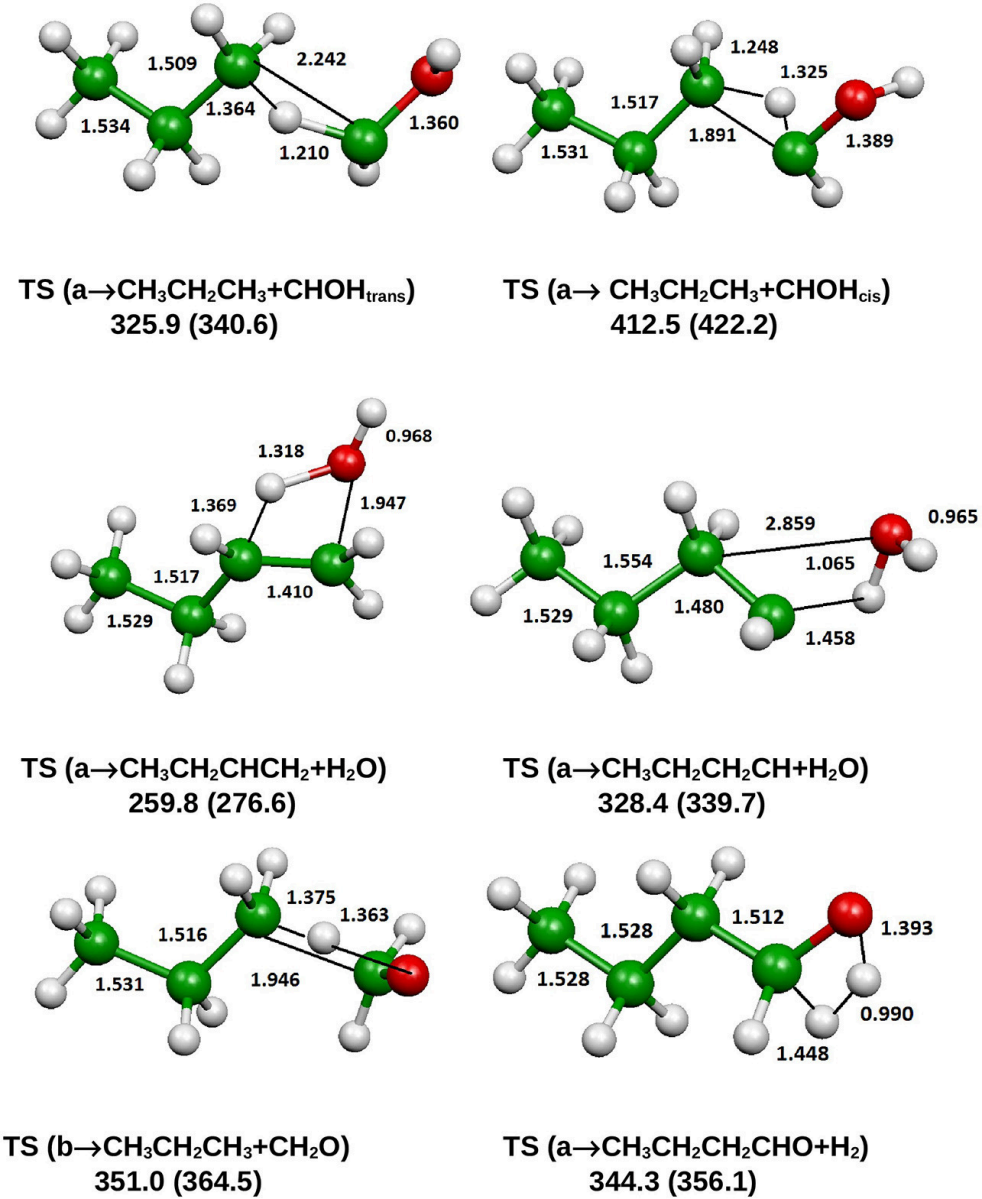
B3LYP optimized geometries (Å and °) and energies, relative to CH_3_CH_2_CH_2_CH_2_OH (a), (kJ/mol) at 298.15 K of saddle points, relevant for the dissociation of 1-butanol; CCSD(T) relative energies are reported in parentheses.

**Figure 10:**
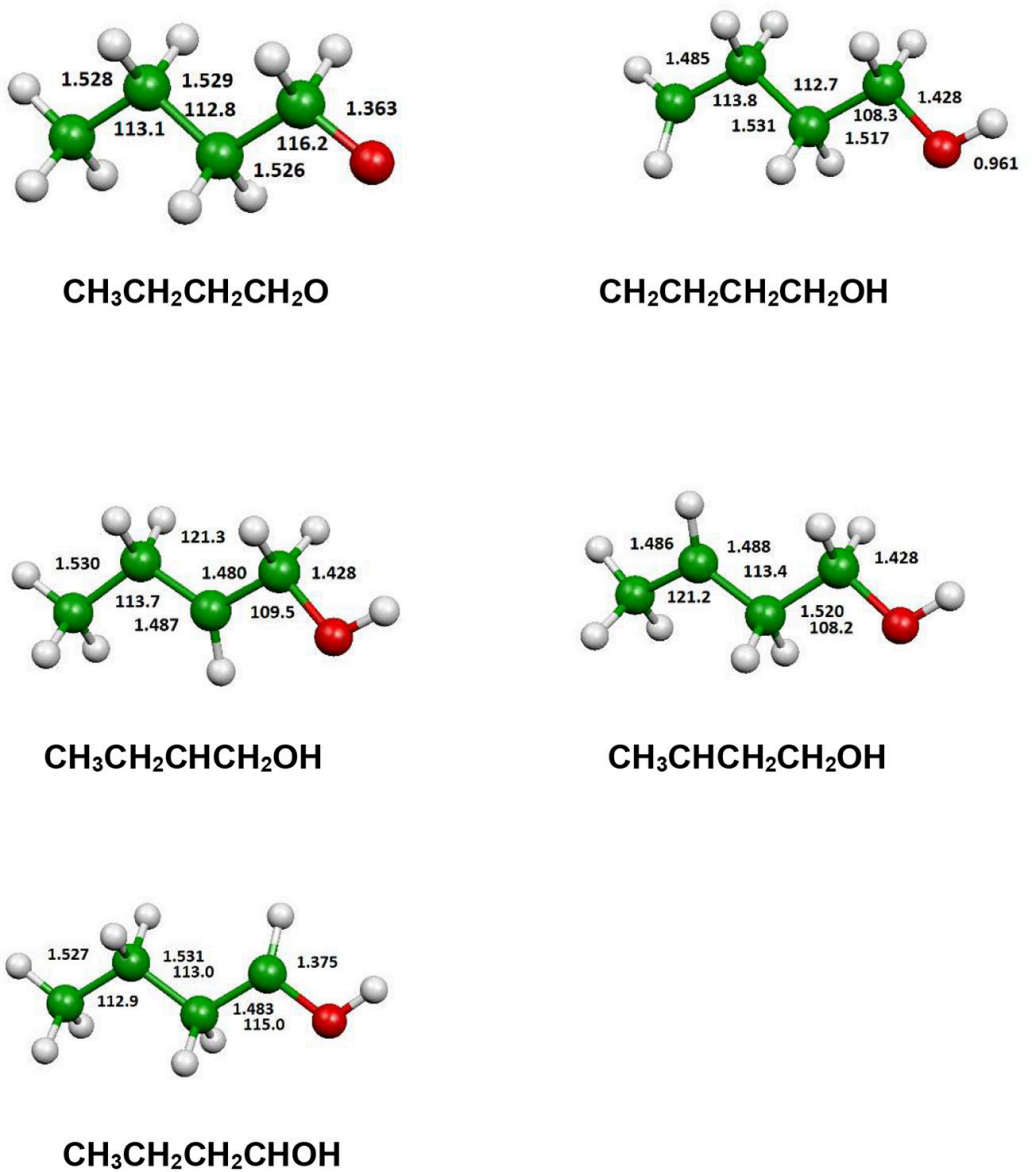
B3LYP optimized geometries (Å and °) of the fragments produced by an hydrogen atom loss process.

The optimized structure of the most stable isomers of 1-butanol is shown in Figure 8, while the optimized structures of the main saddle points localized on the investigated PES are reported in Figure 9 and the optimized structures of the main fragmentation products in the hydrogen atom loss processes in Figure 10. Table 1 reports the enthalpy changes and barrier heights of the main dissociation and isomerization processes for the 1-butanol. From Table 1 we can see that there is a reasonable agreement between B3LYP and CCSD(T) energies. For this reason, we will consider only the more accurate CCSD(T) results. Preliminary partial calculations have been previously reported (Pacifici et al., 2015).

**Table 1:** Enthalpy changes and barrier heights (kJ/mol, 298.15 K) computed at the B3LYP/aug-cc-pVTZ and CCSD(T)/aug-cc-pVTZ levels of theory for selected dissociation and isomerization processes for the system CH_3_CH_2_CH_2_CH_2_OH.

	**ΔH**$\Delta_{298}^{{}^{\circ}}$		**Barrier height**	
	**B3LYP**	**CCSD(T)**	**B3LYP**	**CCSD(T)**
**CH_3_CH_2_CH_2_CH_2_OH (a)**→**CH_3_CH_2_CH_2_CH_2_OH (b)**	0.4	−0.3	10.6	11.3
**CH_3_CH_2_CH_2_CH_2_OH (a)**→**CH_3_CH_2_CH_2_CH_2_OH (c)**	0.1	−0.1	1.3	1.4
**CH_3_CH_2_CH_2_CH_2_OH (a)**→**CH_3_CH_2_CH_3_ +** **CHOH (trans)**	259.4	276.1	325.9	340.6
**CH_3_CH_2_CH_2_CH_2_OH (a)**→**CH_3_CH_2_CH_3_ +** **CHOH (cis)**	276.8	294.3	412.5	422.2
**CH_3_CH_2_CH_2_CH_2_OH (b)**→**CH_3_CH_2_CH_3_ +** **CH_2_O**	39.6	62.0	350.7	364.8
**CH_3_CH_2_CH_2_CH_2_OH (a)**→**CH_3_CH_2_CHCH_2_ +** **H_2_O**	23.4	38.1	259.8	276.6
**CH_3_CH_2_CH_2_CH_2_OH (a)**→**CH_3_CH_2_CH_2_CHO** **+** **H_2_**	59.4	72.8	344.3	356.1
**CH_3_CH_2_CH_2_CH_2_OH (a)**→**CH_3_CH_2_CH_2_CH** ^**…**^ **H_2_O**	333.0	341.8	328.4	339.7
**CH_3_CH_2_CH_2_CH** ^**…**^ **H_2_O**→**CH_3_CH_2_CH_2_CH** **+** **H_2_O**	12.2	9.7		
**CH_3_CH_2_CH_2_CH**→**CH_3_CH_2_CHCH_2_**	−321.7	−313.8	7.3	8.1
**CH_3_CH_2_CH_2_CH_2_OH (a)→CH_3_ + CH_2_CH_2_CH_2_OH**	340.2	369.0		
**CH_3_CH_2_CH_2_CH_2_OH (a)**→**CH_3_CH_2_CH_2_ + CH_2_OH**	318.8	357.3		
**CH_3_CH_2_CH_2_CH_2_OH (a)**→**CH_3_CH_2_ + CH_2_CH_2_OH**	330.5	369.9		
**CH_3_CH_2_CH_2_CH_2_OH (a)→CH_3_CH_2_CH_2_CH_2_ + OH**	366.1	386.6		
**CH_3_CH_2_CH_2_CH_2_OH (a)**→**CH_2_CH_2_CH_2_CH_2_OH + H**	413.0	420.9		
**CH_3_CH_2_CH_2_CH_2_OH (a)**→**CH_3_CHCH_2_CH_2_OH + H**	396.6	410.0		
**CH_3_CH_2_CH_2_CH_2_OH (a)**→**CH_3_CH_2_CHCH_2_OH + H**	401.2	415.5		
**CH_3_CH_2_CH_2_CH_2_OH (a)**→**CH_3_CH_2_CH_2_CHOH + H**	381.6	395.0		
**CH_3_CH_2_CH_2_CH_2_OH (a)**→**CH_3_CH_2_CH_2_CH_2_O + H**	415.1	432.2		
**CH_3_CH_2_CH_3_ + CH_2_O**→**CH_3_CHCH_2_ + CH_3_OH**	29.7	36.0	172.4	179.5
**HCOH (trans)**→**CH_2_O**	−219.4	−214.2	127.3	127.5
**HCOH (cis)**→**HCOH (trans)**	−17.2	−18.2	93.1	94.9

**Figure 11:**
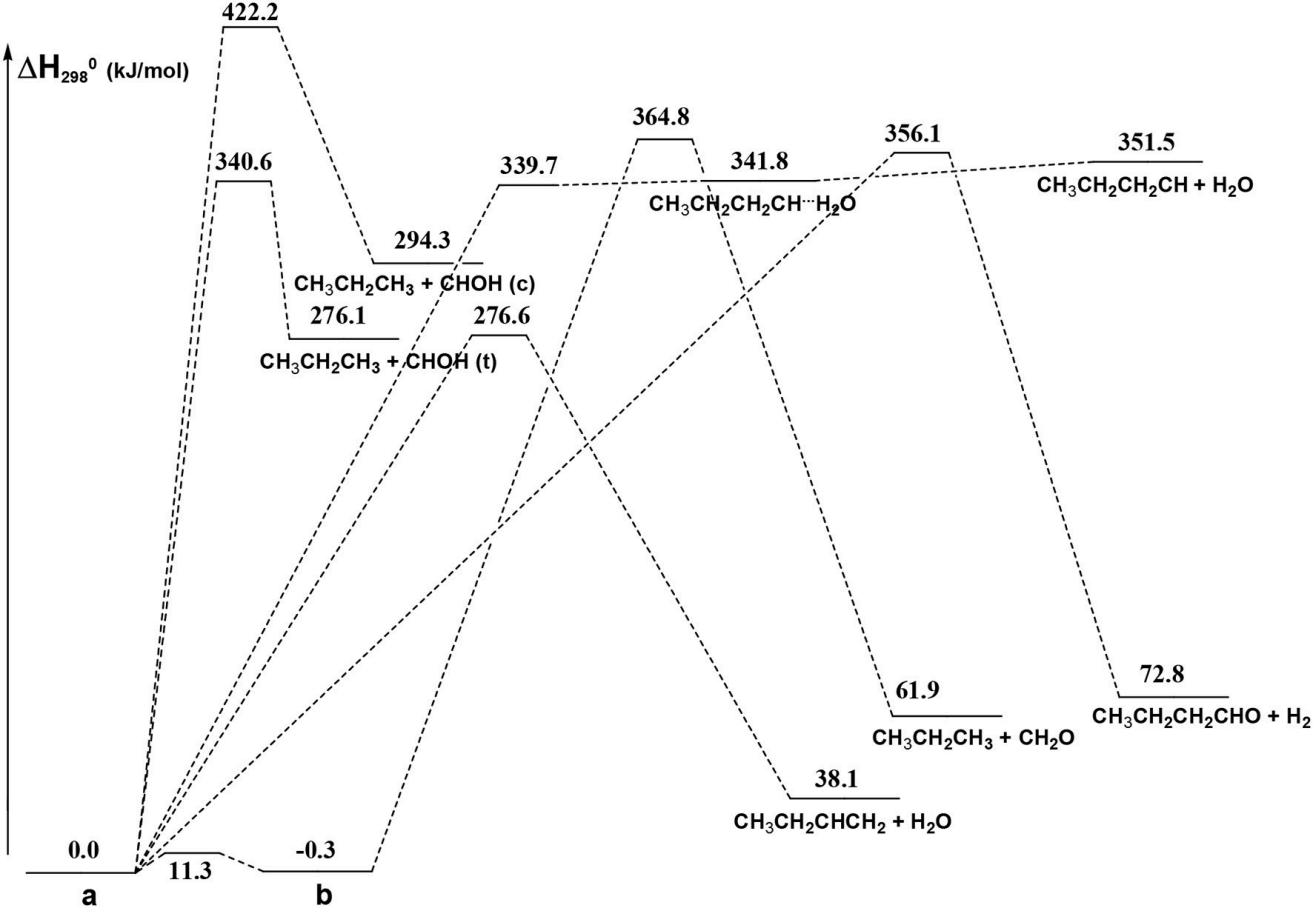
Schematic representation of the 1-butanol dissociation channels showing an exit barrier. For simplicity, only the CCSD(T) relative energies (kJ/mol) are reported.

**Figure 12:**
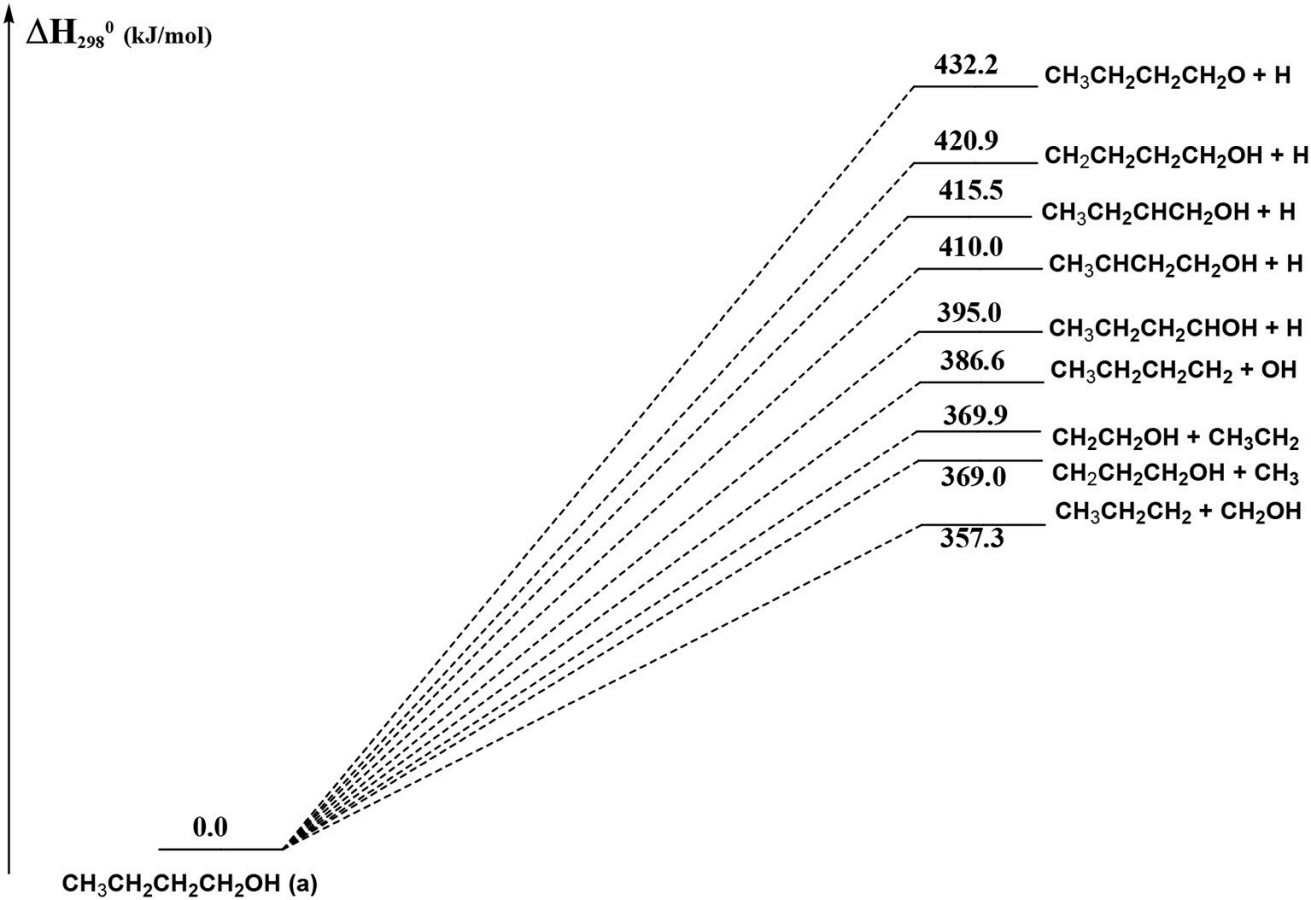
Schematic representation of the 1-butanol dissociation channels which do not show an exit barrier. For simplicity, only the CCSD(T) relative energies (kJ/mol) are reported.

From Figure 8 we can see that we have an isomer (a) which shows a *C*_s_ symmetry and two other isomers, almost degenerate with (a), which show no symmetry. As we can see from Table 1, at B3LYP level (a) is the most stable species, while at CCSD(T) level (b) is the most stable isomer, but only by 0.3 kJ/mol with respect to (a). Being the energy differences among these species below the estimated accuracy of the calculations (±5 kJ/mol), we will refer all the following discussion to the most symmetric species (a). In (a) both the dihedral angles ∠ HOCC and ∠ OCCC are equal to 180.0°, while in (b) ∠HOCC is equal to −65.4° and ∠ OCCC is equal to −62.6° and in (c) ∠ HOCC is 61.6° and ∠ OCCC is 177.6°. The dihedral angles are the main differences among these three isomers and, being the C—C and C—O bonds all single bonds, the isomerization saddles among these species are expected to be very low in energy. As we can see from Table 1, the isomerization of (a) to (c) is almost barrierless, while the isomerization of (a) to (b) shows a barrier of only 11.3 kJ/mol at CCSD(T) level. In Figures 11, 12 we have reported a schematic representation of the main dissociation channels of 1-butanol. For simplicity, only the CCSD(T) energies are shown in the figure. In Figure 11, we have reported the dissociation processes which involve a transition state, while in Figure 12 we have reported the dissociation processes which involve only the breaking of a bond and are, therefore, endothermic and do not show a transition state, since the geometrical rearrangement is not very pronounced. From Figure 11, we can see that 1-butanol can dissociate producing water, molecular hydrogen, formaldehyde and its isomer CHOH, both in the more stable *trans* and in the *cis* structure. All these reactions imply the presence of relatively high transition states. The dissociation of 1-butanol into CH_3_CH_2_CH_3_ and CHOH imply a barrier of 340.6 kJ/mol for the formation of the *trans* species and 422.2 kJ/mol for the formation of the *cis* isomer. Both reactions are endothermic, the first by 276.1 kJ/mol and the second by 294.3 kJ/mol. After formation, CHOH *cis* can isomerize to the more stable (by 18.2 kJ/mol at CCSD(T) level) *trans* species but this reaction shows a barrier of 94.9 kJ/mol, being involved the breaking of a partial double bond. CHOH *trans* can also isomerize to the more stable (by 214.2 kJ/mol) formaldehyde species with a barrier of 127.5 kJ/mol. Formaldehyde can be formed also from 1-butanol, starting from isomer (b). This reaction which is endothermic by 62.2 kJ/mol shows a barrier as high as 365.1 kJ/mol. CH_3_CH_2_CH_3_ and CH_2_O can isomerize to CH_3_CHCH_2_ and CH_3_OH producing methanol. However, this reaction, endothermic by 36.0 kJ/mol, shows a barrier of 179.5 kJ/mol, as we can see from Table 1. The production of molecular hydrogen from 1-butanol is an endothermic reaction (by 72.8 kJ/mol) with a barrier as high as 356.1 kJ/mol. Water can be produced in two different reactions. The first one gives water and the more stable species CH_3_CH_2_CHCH_2_; this reaction is endothermic by only 38.1 kJ/mol and has a barrier of 276.6 kJ/mol. In the other reaction we have the formation of the less stable species CH_3_CH_2_CH_2_CH; this reaction shows a barrier of 339.7 kJ/mol, which is almost equal to the endothermicity of the process.

From Figure 11 we can notice that the main dissociation channel should be the one leading to 1-butene and water since it shows the lowest barrier. Due to the relevance of this point, we decided to perform comparison calculations in order to check the reliability of our results, following also the useful suggestions of the referees. We computed the barrier for the dissociation reaction of (a) into 1-butene and water also at the G3 (Curtiss et al., 1998) and G3B3 (Baboul et al., 1999) level. From Table 1 we can see that the barrier height for this reaction is 259.8 kJ/mol at B3LYP/aug-cc-pVTZ level and 276.6 kJ/mol at CCSD(T)/aug-cc-pVTZ level. This discrepancy is expected since it is well-known that B3LYP usually tends to underestimate the energy of the transition states, although it provides a reasonable estimate of the optimized geometries. This is confirmed by the G3 method which provides for the same reaction a barrier height of 281.1 kJ/mol. At G3B3 level we computed a barrier height of 279.3 kJ/mol. Therefore, the values computed at CCSD(T)/aug-cc-pVTZ, G3 and G3B3 level differ by less than the estimated uncertainty (±5 kJ/mol) in the calculations. The CCSD(T)/aug-cc-pVTZ barrier height computed at the G3 (G3B3) optimized geometry is 279.0 (277.5) kJ/mol, confirming that the B3LYP provides a correct description of the optimized geometries, although it underestimates the energies of the transition states.

In Figure 12 we have reported the main dissociation channels which do not show a transition state. All these reactions are highly endothermic. In particular, the hydrogen atom loss reactions show an endothermicity around or higher of 400 kJ/mol.

The same reaction has been previously investigated by Cai et al. (2012). The agreement between our results and Cai et al. investigation is very good, despite the different methodologies employed. The only significant difference is in the channels leading to HCOH because we considered both the formation of HCOH *trans* and *cis*, while Cai et al. (2012) considered only one species, without specifying which one they investigated.

**Figure 13:**
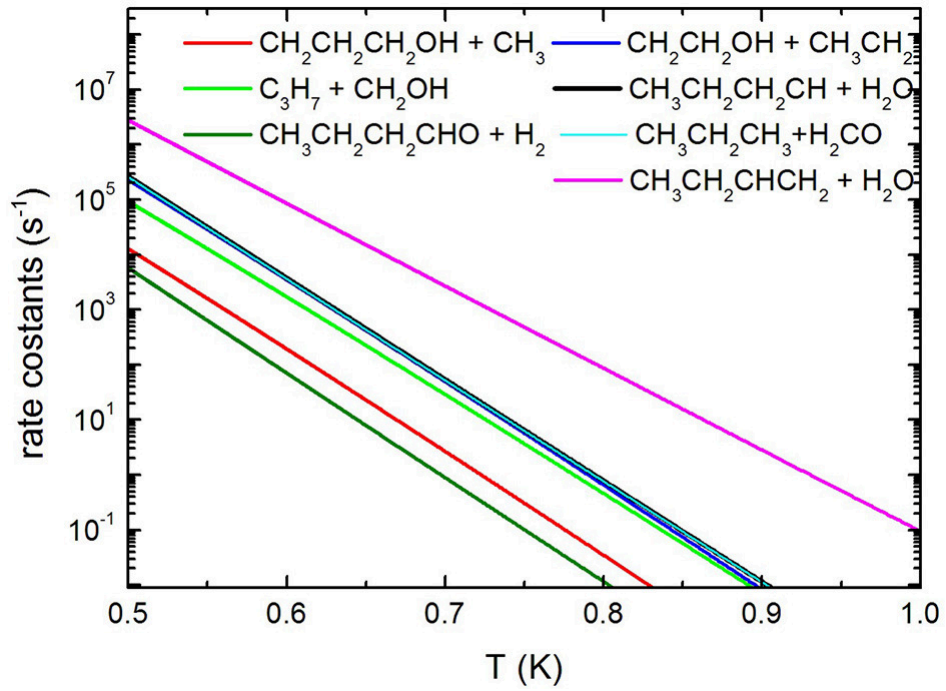
Unimolecular decomposition rate coefficients for the most relevant dissociation channels.

**Figure 14:**
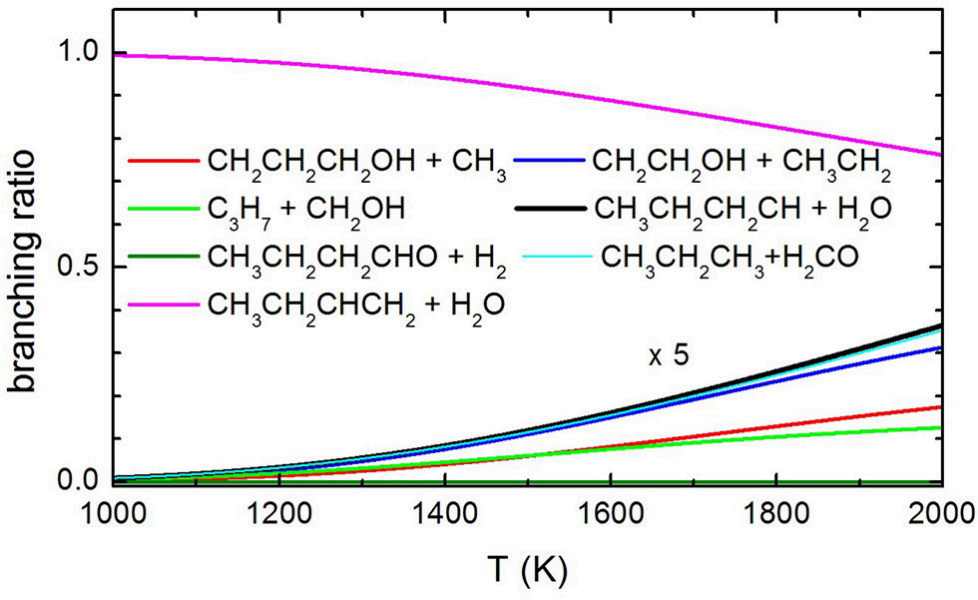
Branching ratio of the most relevant channels as a function of temperature. The branching ratio for the channels other than 1-butene formation with H_2_O elimination are expanded by a factor of 5 to make them visible.

Concerning the kinetics calculations, some details specific to the system considered should be mentioned. It was reasonably assumed that the reaction starts from an equilibrium population of the two 1-butanol conformers. Moreover, in the case of the production of the three interconvertible species CHOH(cis), CHOH(trans), and CH_2_O, it was assumed that an eventual equilibrium was reached between them. As a result, the rate constants for the production of these three species were summed together for each energy and, subsequently, this overall rate constant was partitioned between the three based on the density of states of each species for the particular energy considered. The rate coefficients for the most important channels are reported in Figure 13, while the relative branching ratios for the seven channels actually contributing up to 2,000 K are shown in Figure 14.

It can be seen that the most abundant channel by far is CH_3_CH_2_CHCH_2_ + H_2_O. The reason for this is easily seen to be the fact that the barrier to this channel is the lowest one. Nevertheless, this barrier lies 276.6 kJ mol^−1^ above the reactants and, as a result, the corresponding rate constant becomes relevant only above 1,000 K. Our rate coefficient for this channel is essentially identical to the one calculated by Cai et al. (2012), apart from the highest temperatures, where the rate coefficient seems to increase with a different slope. We suspect that this curvature of their rate constants at high temperatures may be due to the fact that Cai et al. only consider energies up to 600 kJ mol^−1^ while we go up to 1,500 kJ mol^−1^. The next two channels, of similar abundance to each other (but much lower than the first one) are CH_3_CH_2_CH_2_CH + H_2_O and CH_3_CH_2_CH_3_ + CH_2_O (which are not shown by Cai et al. in their Arrhenius plots). Even though the barrier to the first of the two is significantly lower than the barrier to CH_2_O formation, it should be remembered that the latter channel is in equilibrium with the formation of the CHOH (cis and trans) products, as CHOH isomerizes without losing energy. As the formaldehyde product lies much lower in energy than both CHOH species, it is almost exclusively favored at equilibrium and, thus, all rate constants leading to CHOH or CH_2_O essentially are rate constants for formaldehyde formation. In particular, the barrier leading to CHOH (trans) from the (a) conformer of butanol is essentially identical to the barrier leading to CH_3_CH_2_CH_2_CH + H_2_O and this explains the similar abundance of the two channels.

The next highest channel (of very similar abundance to the previous ones) is the formation of CH_2_CH_2_OH + CH_3_CH_2_, followed by the CH_2_OH + CH_3_CH_2_CH_2_ one. Even though it is the second of these two channels that has the lowest energy barrier of the two, the first one is augmented by an increased density of states caused by low-frequency vibrational modes, i.e., an entropy effect. This inversion of the rate constants with respect to the potential barriers is also seen in the rate constants of Cai et al. For the same reason, the CH_2_CH_2_CH_2_OH + CH_3_ channel, even though it has an energy barrier very similar to the CH_2_CH_2_OH + CH_3_CH_2_ channel, is penalized by the high rotational constant of the methyl radical which drastically reduces its density of states. Again, this is also precisely the effect seen in the Cai et al. rate constants. The effect is seen even more clearly for the butanal + H_2_ production channel (which is not considered by Cai et al.), whose rate constant is noticeably even lower than that of CH_3_ production.

It is to be noted that the rate constants of Cai et al. (2012) for the last three channels mentioned (which have monotonic reaction paths), although very similar to ours, are consistently somewhat higher. As our rate constants have been computed variationally (choosing the minimal rate constant among various candidate transition states), we feel that such a difference may be due to an incomplete sampling of the configuration space by the authors.

Finally, the other channels we have computed (pertaining to OH and H elimination from the original butanol molecule) only play a minor role in the kinetics and we have deemed their rate constants undetectable by the present experiment. Therefore, the assumption used in the analysis of our experimental results is fully supported by the present calculations.

## Conclusions

The present experimental results clearly demonstrate that butene is an important pyrolysis product under the experimental conditions of our experiments. These results are confirmed by the theoretical calculations of the decomposition rate coefficients which identify 1-butene as the most important product in the temperature range spanned in our experiments. In addition to that, there is no experimental evidence that 1-butanal is formed by elimination of molecular hydrogen. Considering that butanal has a parent peak with a significant intensity at 70 eV, were it formed we should have seen it. Finally, our results indicate that methyl elimination is also occurring. As for the other channels, we have a clear indication that C-C bond breaking channels are occurring, but we are unable to quantify their yields as they interfere with each other because of dissociative ionization. Nonetheless, it is clear from the trend reported in Figure 14 that the extent of pyrolysis and the impact of the other channels is increasing with the temperature.

Our theoretical investigation is in line with previous characterization and is in agreement with the experimental results. The H-elimination channels have been reported here for the first time, but, as expected because of their high energy levels, they do not contribute significantly to the process.

## Data Availability

The raw data supporting the conclusions of this manuscript will be made available by the authors, without undue reservation, to any qualified researcher.

## Author Contributions

DoS, NB, SF, and CN performed the experiments and the experimental data analysis. MR and LP performed the electronic structure calculations. DiS and NF performed the kinetics calculations. The manuscript was written by NB, DiS, DoS, and MR.

### Conflict of Interest Statement

The authors declare that the research was conducted in the absence of any commercial or financial relationships that could be construed as a potential conflict of interest.

## Footnotes

^1^https://webbook.nist.gov/cgi/cbook.cgi?ID=C123728&Units=SI&Mask=200#Mass-Spec

^2^https://webbook.nist.gov/cgi/cbook.cgi?ID=C71363&Units=SI&Mask=200#Mass-Spec

^3^https://webbook.nist.gov/cgi/cbook.cgi?ID=C106989&Units=SI&Mask=200#Mass-Spec

^4^https://physics.nist.gov/cgi-bin/Ionization/table.pl?ionization=C4H8xx0
